# Is Habituation Measurable in Lumpfish *Cyclopterus lumpus* When Used as Cleaner Fish in Atlantic Salmon *Salmo salar* Aquaculture?

**DOI:** 10.3389/fvets.2019.00227

**Published:** 2019-07-09

**Authors:** Fredrik R. Staven, Jarle T. Nordeide, Albert K. Imsland, Per Andersen, Nina S. Iversen, Torstein Kristensen

**Affiliations:** ^1^Faculty of Biosciences and Aquaculture, Nord University, Bodø, Norway; ^2^Aqua Kompetanse AS, Flatanger, Norway; ^3^Akvaplan-niva Iceland Office, Kópavogur, Iceland; ^4^Department of Biological Sciences, University of Bergen, Bergen, Norway; ^5^Namdal Rensefisk, Flatanger, Norway

**Keywords:** lumpfish, Atlantic salmon, interaction, stress, habituation

## Abstract

To investigate how lumpfish interact in Atlantic salmon aquaculture, physiological stress responses and changes in behaviour were analysed in experienced and naive lumpfish. Experienced lumpfish (30.2 ± 7.93 g, mean ± SD) coexisted with a commercial scale production unit of Atlantic salmon (1258.5 ± 152.12 g) for 30 to 60 days, while naive lumpfish (38.2 ± 12.37 g) were kept with conspecifics only. Ten trials from each background were tested. For each trial, 10 lumpfish were tagged and transferred to a video monitored experimental tank (2 × 2 × 0.7 m). In each trial, swimming behaviour was mapped for all lumpfish every 60 s in 20 min, 10 min before, and 10 min after the introduction of four Atlantic salmon. Naive lumpfish expressed significantly increased burst swimming activity and maintained longer interspecific distance to Atlantic salmon in comparison with experienced fish. In addition, mean plasma cortisol levels were significantly elevated in naive fish after exposure to Atlantic salmon. We argue that naive lumpfish expressed innate physiological and behavioural stress responses during first encounter with Atlantic salmon, while reduced responses in experienced individuals indicated habituation. The effect from behavioural and physiological stress in newly deployed naive lumpfish–before and during habituation–should be taken account for when lumpfish are introduced in commercial sea cages to improve welfare for the species. In addition, we suggest that habituation could be applicable during the rearing phase to moderate the transition from a simple tank environment with conspecifics only to interspecies interaction with Atlantic salmon in sea cages.

## Introduction

The main limitation for further growth in Norwegian aquaculture of Atlantic salmon is the ectoparasitic sea lice copepods *Lepeophteirus salmonis* Krøyer, 1837 and *Caligus elongatus* von Nordmann 1832. Sea lice have a negative impact on animal welfare, economic costs, public reputation, and on wild populations of salmonids (1–3). A complete method for sea lice removal has remained yet to be discovered, and the aquaculture industry has been dependent on a toolbox with multiple treatment methods, some more successful than others (4). In contrast to chemotherapeutic and mechanical treatments, the use of cleaner fish is a considerably less stressful and a more sustainable delousing method concerning mortality and welfare of the farmed Atlantic salmon (5–7). On the other hand, animal welfare of cleaner fish has become a new moral challenge for the industry, as uncontrolled loss of cleaner fish, assumed dead during the production period of Atlantic salmon could be considered unethical use of live animals.

Cleaner fish are usually small fish species from different taxa that remove ectoparasites, dead skin or mucus from a larger host “client” fish (8–10). The interspecific relationship resembles a unique mutualistic interaction that benefits both parts. The cleaner collects an ectoparasitic food item from a potential killer, while the client is relieved from parasitic pressure without falling for the temptation of eating the cleaner fish (9, 11). In Norway, four endemic species of cleaner wrasses have been captured and implemented in aquaculture (12–16). In recent years, the lumpfish (or lumpsucker) *Cyclopterus lumpus* Linnaeus, 1758 has been systematically tested as a possible sea lice grazer (17–19), especially at cold sea water temperatures, where the use of wrasse is ineffective (20). Studies conducted in Northern Norway found significantly reduced sea lice infestations on Atlantic salmon when reared together with 5, 10, or 15% lumpfish, with removal of up to 93–97% adult female stages of sea lice in comparison with control groups (17–19, 21). From 2012 to 2016, the production of hatched, reared, and deployed juvenile lumpfish increased from a few thousands to 30 million, which by 2019 made it the second most reared fish species in Norway (22).

During the transition from hatchery to sea cages, lumpfish are put through a series of stressors, including the introduction to Atlantic salmon. While domesticated Atlantic salmon are presumed uninterested in other food items than pellets, their carnivorous nature include consumption of fish of similar size as juvenile lumpfish (23). Nature selects swiftly upon individuals with deficient innate escape performance during predatory encounters, and even obligate cleaner fish, including bluestreak cleaner wrasse *Labroides dimidiatus* Valenciennes, 1839 express such performance when interacting with predatory clients, especially during first encounter (24). Prey fishes first encounter with carnivores triggers behavioural rapid burst swimming (25–27) and predatory avoidance (28–30). First encounters may also lead to physiological responses with increased plasma cortisol levels which promote an increased energy budget from plasma glucose to use for escape and overall vigilance (31, 32). In other teleosts, exposure to stressors which cannot be avoided, has previously revealed behavioural changes in place preference, locomotion style and swim speed (33), as well as impacts on cognitive decision-making, which could result in less time spent on feeding and resting (29, 34), and in the case for aquaculture cleaner fish, less time spend grazing sea lice. Repeated stressors have also shown to desensitize fish and moderate neuroendocrine responses to similar stressors (35), commonly referred to as habituation (36). Habituation, measured as desensitized neuroendocrine responses, for example reduced cortisol secretion during repeated predatory interactions, has previously been demonstrated in model species zebrafish *Danio rerio* Hamilton, 1822 (37). Habituation is considered adaptive when the individual cease to respond to repeated stimuli that has no direct consequence (38). In the light of an increased use of lumpfish in aquaculture, information about the species ability to cope and adapt to the presence of Atlantic salmon is of interest, and available for investigation.

The aim of the study was to examine how lumpfish with experience from living in an open sea cage with farmed Atlantic salmon for 30–60 days expressed measurable changes in behaviour and physiological responses compared to first encounter for naive lumpfish. This emphasises a relatively broad question whether behavioural and physiological responses are affected by interactions with client fish, in this case Atlantic salmon. It was hypothesized that experienced lumpfish would express habituation when reintroduced with Atlantic salmon measured as (1) lower swimming activity, (2) shorter interspecies distance, and (3) lower plasma cortisol levels compared to naive lumpfish.

## Materials and Methods

### Ethical Statement

The study was conducted according to the Animal Welfare Act (LOV-2009-06-19-97) and the Norwegian law on Regulation of Animal Experimentation (FOR-1996-01-15-23). Handling of live fish was managed by personnel with FELASA-C course, based on the policies by the Federation of European Laboratory Animal Science Association. Lumpfish and Atlantic salmon used during the field experiment were assigned to project FDU 7835, accepted by the Norwegian Food Safety Authority under the regulation of the Research Animal Act (FOR-2015-06-18-761).

### Lumpfish

All lumpfish used during this study originated from gametes extracted from sexually mature wild individuals (7 males and 24 females) caught with gill nets in Flatanger (64°30′20.4″N, 10°50′40.8″E), Norway in September 2015. Fertilized eggs were distributed between Namdal Rensefisk in Flatanger and Nordland Rensefisk at Lovund (66°22′01.1″N, 12°22′36.4″E). At Namdal Rensefisk, incubation started during the 1st week of October, while at Lovund, incubation started during the 1st week of November. Larvae hatched from November 15 at Namdal Rensefisk and from December 18 at Lovund Rensefisk. At both locations, feeding consisted of dry feed pellets. During the first 2 months, all lumpfish were fed with Gemma Micro 150 and 300 (Skretting, Stavanger, Norway) and Gemma Wean Diamond, 0.5 mm (Skretting, Stavanger, Norway). In the next months and until departure, lumpfish were gradually fed with Gemma Diamond 0.8, 1.5, Silk 1.5, and 1.8 mm (Skretting, Stavanger, Norway) following feeding recommendations from Skretting AS. All lumpfish were reared in circular green tanks measuring 2.5 m^3^ and later moved to 5.5 m^3^ tanks during the last month prior to departure. At both locations, daily monitored oxygen saturation was above 80%. At Lovund Rensefisk, mean water temperature was 8.0°C, S.D. ± 0.95, with T_max_ 9.5°C and T_min_ 7.5°C. At Namdal Rensefisk, mean water temperature was 7.4°C, S.D. ± 0.95, with T_max_ 9.6°C, and T_min_ 6.3°C. All lumpfish, both at Nordland rensefisk and Namdal rensefisk, were manually vaccinated with AMarine micro 4–2® (Pharmaq, Overhalla, Norway) during the 1st week of June.

### Atlantic Salmon

All Atlantic salmon originated from the AquaGen strain, hatched and reared at Flatanger Settefisk, Trøndelag, Norway. Smolt were deployed in October 2015 and reared in sea cages, 135 m in diameter, until the experiment started at Raudøya (64°21′59.5″N, 10°26′40.9″E) on August 2, 2016. Atlantic salmon (*n* = 80) later selected to the experiment, had a mean weight of 1258.5 g, S.D. ± 152.12 and were collected from sea cage number 4, thus unfamiliar with experienced lumpfish used in the experiment.

### Pre-experiment Preparations for Experienced Lumpfish

Lumpfish referred to as experienced lumpfish, were transported on June 5 from Nordland Rensefisk to Raudøya and deployed in sea cage 5. On arrival, a random sample of experienced lumpfish (*n* = 30) had a mean weight 30.2 g, S.D. ± 2.50. Experienced lumpfish coexisted with Atlantic salmon from June 5 and until the first trial was conducted on August 2, 2016. Each sea cage had cleaner fish shelters preinstalled, 8 m deep spread along 30 m of rope. Experienced lumpfish were fed daily with 2% of total biomass with Lumpfish Grower 2.0 mm pellets (Biomar, Karmøy, Norway).

### Pre-experiment Preparations for Naive Lumpfish

Naive lumpfish (*n* = 100) were transported from Namdal Rensefisk to Raudøya and kept in a 2 ×2 ×1.5 m tank at the feed barge, 200 m northwest from sea cage 4, from July 27 and throughout the trial period until September 6. Naive fish had no previous experience with Atlantic salmon. On arrival, naive fish had a mean weight of 38.2 g, S.D. ± 3.91. Naive lumpfish were fed daily with 2% of total biomass with Lumpfish Grower 2.0 mm pellets (Biomar, Karmøy, Norway). A Metabo® 24 V immersion pump (Metabo, Nürtingen, Germany) provided 7,000 l/h unfiltered seawater from 3 m depth. Environmental data were logged every tenth min using a SD 204 CTD (SAIV, Bergen, Norway). From July 27 to September 3, mean dissolved oxygen was 7.05 mg/L. Mean water temperature was 14.8°C with T_max_ 16.17°C, and T_min_ 13.54°C. Mean conductivity was 40.47 uS/cm and mean fluorescence was 5.71 ug/L.

### Experimental Design

From August 2 and until September 3 in 2016, 20th trials were conducted, including 10 trials with interaction between experienced lumpfish and Atlantic salmon, and 10 trials with interaction between naive lumpfish and Atlantic salmon. Each trial consisted of 10 new lumpfish and four new Atlantic salmon, and no fish was included in more than one trial, which makes each trial a true replicate. Each trial was recorded at the feed barge in a green 2 ×2 ×0.7 m experimental tank with a tarpaulin attached 2 m above to give even light in the tank. To reduce diurnal variation in hormonal outputs (39), all experimental activity was carried out at daytime between 12:00 p.m. and 14:00 p.m. Experienced lumpfish were collected from sea cage 5, while naive fish where collected from the nearby tank at the feed barge. Experienced and naive lumpfish were kept in different environments prior to the experiment due to practical limitations and reason that all sea cages were in full production, accommodated by farmed salmon. Prior to each trial, all 10 lumpfish were lightly sedated with 0.1 ml l^−1^ Benzoak Vet (ACD Pharmaceuticals, Leknes, Norway) and tagged with numbered Petersons discs to identify each fish during video recordings. After tagging, fish were taken off feeding and acclimated for 48 h before the trial was ready for video recording of behaviour and measurements of physiological stress responses.

### Video Recordings

Trials were video recorded with GoPro Hero 3^+^ cameras (Gopro™, California, USA) attached in each corner of the experimental tank, in addition to a camera above the tank. Video was recorded in Full HD at 60 frames per second with ultra-wide field of view in the tank, and medium field of view above the tank. Ten minutes prior to filming, water circulation was stopped to increase visibility in the tank. Next, cameras were synchronously started with a GoPro Wi-Fi remote to record behaviour among conspecifics of lumpfish in the tank. After 10 min, four Atlantic salmon were carefully added to the tank by hand net from behind a cover. To avoid burst swimming after handling, Atlantic salmon were lightly sedated with 0.05 ml l^−1^ dosage of Benzoak Vet (ACD Pharmaceuticals, Leknes, Norway) for 5 min. The video recordings continued for 10 min to observe the behavioural interaction between lumpfish and Atlantic salmon. A total of 20 min was video recorded during each trial, in addition to 25 min of delay before each trial was ended. This was done to ensure that cortisol reached closer to peak level after 45 min from introduced stressor, as previously observed by Iversen et al. (40). A dosage of 3 mg l^−1^ Aqua calm® (Western Chemical Inc., Canada) with cortisol blocking properties, was added to the experimental tank. After 5 min, lumpfish were hand netted and humanely euthanized with a blow to the head. Oxygen and temperature were logged in the experimental tank after each trial using a SD 204 CTD (SAIV, Bergen, Norway). From 20 trials, mean oxygen was 7.68 mg/L and mean water temperature was 14.7°C.

### Data Collection

#### Physiological Data

Blood from euthanized lumpfish was collected after each trial. Blood was collected from the heart ventricle using a 0.33 ×12.7 mm syringe (BD Micro-fine®) containing anticoagulating heparin before weight and length was registered. Blood were put in Eppendorf tubes and centrifuged at 6,000 rpm (rounds per minute) for 5 min in a Mini Star centrifuge (VWR™, UK). After centrifugation, plasma was separated with a pipette, transferred to a 1.8 ml Nunc Cryo Tube® and stored at −30°C. Samples were later analysed for plasma cortisol measurements at Nord University in Bodø, Norway using Radioimmunoassay, previously described by Iversen et al. (41). As a tracer, [1, 2, 6, 7, −3H] Cortisol (Amersham plc, Oslo, Norway), treated with 250 mCi (9.25 MBq) and diluted in 25 mL of absolute alcohol (Activity of about 10 mCi/mL) was used. Hydrocortisone (H 4001, Sigma-Aldrich, Oslo, Norway) was applied to produce a standard range from 0 to 137.5 nmol/L. The antibody was obtained from Sheep Anti-Cortisol, code: S020 (Guildhay Ltd, Surrey, UK). Samples were incubated at 1–2°C for 24 h before centrifuged with a Haraeus sepatech Omnifuge 2.ORS radius 154 mm, rotor 3,360. Antibody-antigen complex was counted in a scintillation counter type Packard Tri Carb 1900 TR. The sensitivity in the assay was 1.68 nmol/L. Samples under “detection limit” were set equal to the sensitivity of the assay. Intra-assay was below 10% and inter-assay was 12.5% at 80 nmol/L. NSB ranged from 2.1 to 4.8% of the total activity. Previously executed recovery tests at the laboratory of Nord University gave the following results: Measurement of 4, 17, 34, and 69 nmol/L radiolabelled cortisol with added plasma, showed a recovery of 90, 94, 96, and 95%.

#### Behavioural Data

In the experimental tank, swimming activity was registered once every minute for each lumpfish, 10 min before and 10 min after the introduction of Atlantic salmon. Swimming activity was categorized based on previous work by Tully et al. (16) and Imsland et al. (19), with distinguishable locomotion separated into scores (Table 1). Interspecific distance between each lumpfish and the nearest Atlantic salmon was measured using ImageJ2 (42) on still photos from video of the experimental tank in 2D perspective. A line calibrated against the 20 ×20 cm grids in the bottom of the experimental tank was drawn from between the eyes of the lumpfish to between the eyes of the nearest Atlantic salmon.

**Table 1:** Classification of lumpfish swimming activity based on distinguishable locomotion.

**Score**	**Swimming activity**	**Description**
4	Burst	Rapid acceleration in any direction
3	Normal	Locomotion between hovering and burst swimming activity
2	Hovering	Hovering performance with no horizontal or vertical motion
1	Attached	Attached to substrate with sucker disc

### Statistics

All statistical work was computed with R software™ R.3.2.2 (43). A chi-square test was used to compare observed changes in counts of swimming activity between trials of naive and trials of experienced lumpfish. A Shapiro–Wilk test (44) was used to test normality of distributions for both physiological and behavioural data. For comparison of plasma cortisol levels, a Student's *t*-test was used when normality and Levene's *F*-test assumption of homogeneity in variances between populations were met. A Wilcoxon rank sum test was used on non-parametric independent data of naive and experienced trials when these assumptions were not met. A significance level of α = 0.05 was used.

## Results

### Swimming Activity

Counts of the swimming activities “burst” and “hovering”—but not “normal” and “attached”—differed significantly between naive and experienced lumpfish (Table 2). First trial with naive lumpfish and first trial with experienced lumpfish were not included in behavioural analysis due to low visibility in the experimental tank. Main alteration was observed in trials (*n* = 9) with naive fish, where counts of “burst” swimming activity, increased from 9 to 299 after the introduction of Atlantic salmon in the experimental tank, compared to from 5 to 27 in trials (*n* = 9) with experienced lumpfish. Moreover, counts of “hovering” decreased in naive lumpfish, whereas counts of “hovering” increased in experienced fish from before to after Atlantic salmon were introduced (Table 2).

**Table 2:** Counts of swimming activity among replicates (*n* = 9) of naive and replicates (*n* = 9) of experienced lumpfish.

**Swimming activity**	**Naive**		**Experienced**		**Chi-Square values**		
	**Before**	**After**	**Before**	**After**	**χ^2^**	**df**	***P*-value**
Burst	9	229	5	27	8.047	1	0.005
Normal	526	403	606	507	0.967	1	0.325
Hovering	180	129	149	234	25.673	1	<0.001
Attached	185	139	130	112	0.641	1	0.423

*Counts were done of all 10 lumpfish every min in each trial, 10 min prior to introduction of Atlantic salmon in the experimental tank and 10 min after the introduction. Difference in swimming activity before and after the introduction of Atlantic salmon in both naive and experienced lumpfish, was tested using Chi-square tests*.

**Figure 1:**
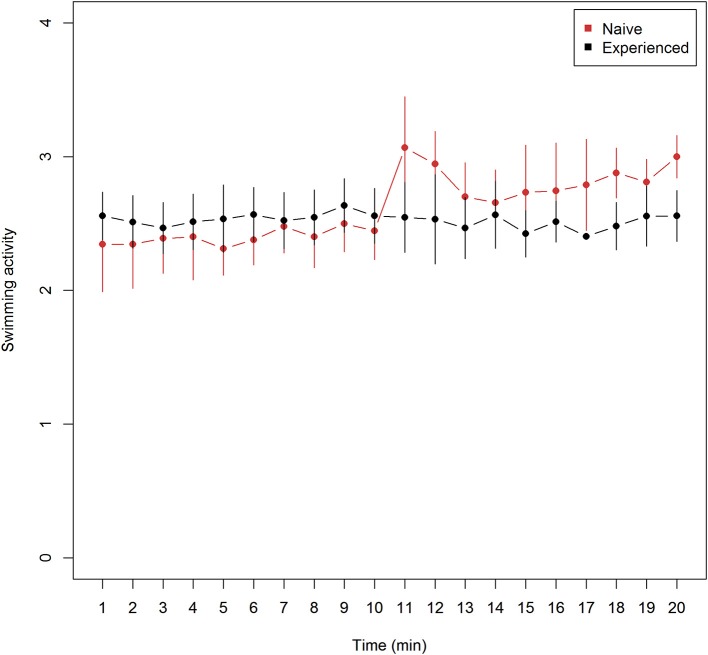
Swimming activity (mean ± 95% confidence intervals) for naive (*n* = 9) and experienced (*n* = 9) trials of lumpfish during 20 min of video observations. Swimming activity include mean ranked observations from the 4 categories (1) attached, (2) hovering, (3) normal swimming, and (4) burst swimming while time show the duration of video recordings −10 min without and 10 min with Atlantic salmon interaction. The Atlantic salmon were added to the tank between 10 and 11 min. While mean values of swimming activity among experienced lumpfish remained unchanged, mean values of naive swimming activity increased significantly at 11 min (Two-sided Wilcoxon rank sum test, *W* = 65, *p* < 0.033).

Mean (± 95% confidence intervals) swimming activity observed every minute from trials of naive (*n* = 9) and experienced (*n* = 9) lumpfish revealed lower means at all observations among naive trials prior to Atlantic salmon introduction and higher means after, in comparison with experienced trials (Figure 1). Mean swimming activity in naive fish increased from the last observation before (10 min), to the first observation after (11 min) after the introduction of Atlantic salmon (Figure 1). (Wilcoxon rank sum test, *W* = 65, *p* = 0.033), and confidence intervals did not overlap at 18 and 20 min into the experiment (Figure 1) while overlapping on the remaining.

### Interspecies Distance

**Figure 2:**
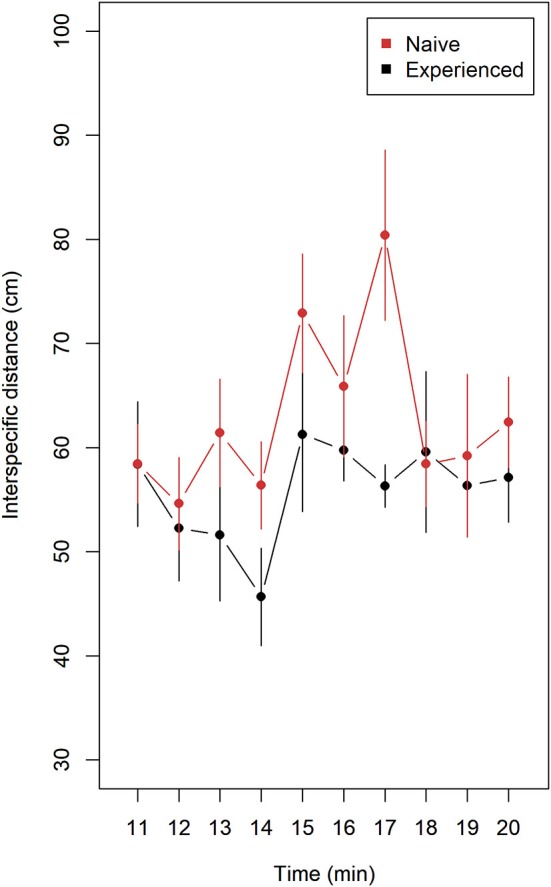
Interspecific distance (mean ± 95% confidence intervals) for naive (*n* = 9) and experienced (*n* = 9) replicates of lumpfish during 10 min of interaction with Atlantic salmon. Distance (cm) was measured between each lumpfish and the nearest Atlantic salmon using ImageJ. Each replicate contained 10 lumpfish and 4 Atlantic salmon. Naive fish tended to keep a longer distance to nearest Atlantic salmon. A significant difference was observed after pooling means of interspecific distance from trials (*n* = 9) of naive and trials (*n* = 9) of experienced lumpfish (Wilcoxon rank sum test, *W* = 79, *p* = 0.028).

Naive compared to experienced lumpfish tended to keep longer distance to Atlantic salmon (Figure 2). Mean values with 95% confidence intervals did not overlap at 14 and 17 min and overlapping on the remaining minutes. A significant difference was observed after pooling means of interspecific distance from trials (*n* = 9) of naive and trials (*n* = 9) of experienced lumpfish (Wilcoxon rank sum test, *W* = 79, *p* = 0.028).

### Plasma Cortisol

**Figure 3:**
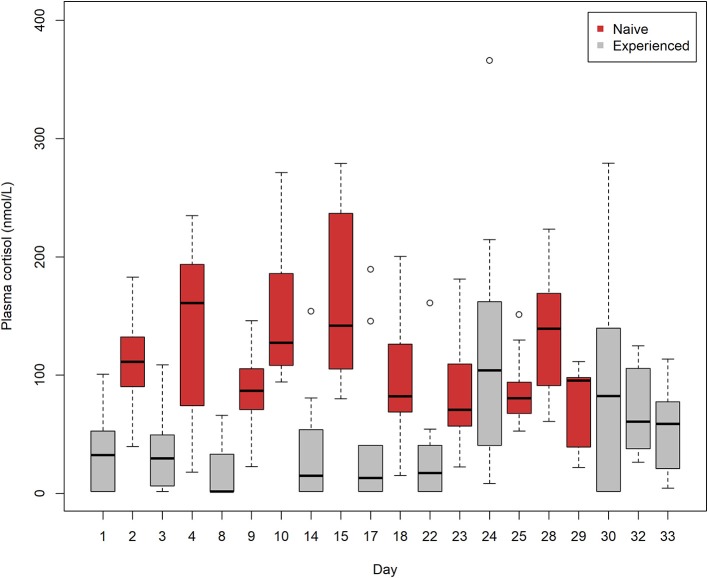
A Box-and-Whiskers plot showing plasma cortisol levels in 10 trials of naive lumpfish and 10 trials of experienced lumpfish after interacting with Atlantic salmon. Trials (*n* = 10) of naive lumpfish had significantly elevated cortisol levels in comparison with trials (*n* = 10) of experienced lumpfish after calculating the mean from each of the 10 trials from each of the two groups of lumpfish (Student's *t*-test, *t* = 3.67, *p* = 0.001, d.f. = 18). Trials were conducted from August 2 to September 3. Plasma cortisol (nmol/l) was measured using radioimmunoassay (RIA).

Plasma cortisol levels in naive lumpfish trials accounted for 8 of the top 10 highest median and mean values out of 20 trials of naive and experienced lumpfish (Figure 3). Moreover, trials (*n* = 10) of naive lumpfish had significantly higher cortisol values in comparison with trials (*n* = 10) of experienced lumpfish after calculating the mean from each of the 10 trials from each of the two groups of lumpfish (Student's *t*-test, *t* = 3.67, *p* = 0.001, d.f. = 18).

## Discussion

### Swimming Activity

Naive lumpfish increased burst swimming activity and interspecies distance to Atlantic salmon, revealed that innate escape behaviour is present and lasting during first interaction, even if lumpfish is considered a facultative cleaner fish which graze ectoparasitic sea lice in salmon aquaculture. This increased vigilance during first encounter with a client fish is similar to observations of obligate cleaner fish (24, 45), Thus, the process of habituating to stressors in an unfamiliar environment with Atlantic salmon before ideal grazing of sea lice occur, require additional learning through perception and repeated interactions to memorize and separate harmless stimuli from harmful ones (46). Prey fish escape behaviour from predators is in many ways a most fundamental defensive adaptation to avoid death and increase individual fitness (47). In nature, juvenile lumpfish have been observed to quickly detach from substrates and flee when a predator fish approached (48), which coincided with observation on naive fish swimming behaviour in the current study. A rapid innate escape response from environmental stressors has also been observed in larval lumpfish, and increased swimming activity as a measurement of escape behaviour is without difficulty applied to the species (26, 49).

Escape behaviour and burst swimming activity are both energetically costly (50) and affects the overall fitness regarding time spent on other tasks (51). In nature, lumpfish are exposed to different stimuli that trigger antipredator behaviours at daily basis, and the ability to habituate and quickly learn how to distinguish danger from safety is essential to adapt and cope in a changing and complex environment (52, 53). Experienced lumpfish that coexisted with Atlantic salmon did not significantly change their swimming activity from the moment of reintroduction in the experimental tank, and the previous experience from interspecies interaction in commercial sea cages appeared to have impacted their behaviour, to the presence of salmon. Predator recognition from sensory cues, including vision, the lateral line system and olfaction, require experience through the ontogeny of fish (54–56). Yet, many species of fish have appeared to be predisposed to certain visual predator cues including shape, colour and size of the body as well as mouth structure (53, 57, 58). When considering the origin of escape behaviour in lumpfish, it should be noticed that the obligate cleaner fish bluestreak cleaner wrasse *L. dimidiatus* have shown to express similar fast-start escape behaviour during hit and runs after intentionally consuming nutrient rich mucus from the skin of a client fish, before being chased away (24). It is uncertain if lumpfish sometimes cheats and eat mucus from Atlantic salmon epidermal skin, and observations on such behaviour would only partly explain why, as ectoparasites are only fractions of the species food types (21) in comparison to obligate cleaner fish (9).

### Physiological Stress Response

In trials with naive lumpfish, plasma cortisol measurements were similar to measurements observed after exposing lumpfish to crowding stress, by draining the tank environment (40), and after exhaustive chasing of lumpfish, also in a tank environment (59). Iversen et al. (40) found that plasma cortisol elevated to 200 nmol l^−1^ 1 h after exposure the stressor, while Hvas et al. (59) found mean plasma cortisol elevations of 150.9 nmol l^−1^. The present study observed a mean value of 114.94 nmol l^−1^ 45 min after the introduction of Atlantic salmon in trials with naive fish. These values interrelated with the time perspective, which indicated that naive lumpfish showed a strong primary stress response due to the presence of Atlantic salmon. Publications on habituation in lumpfish are absent, yet similar studies on other species have observed habituation in prey zebrafish *D. rerio* after repeated interactions for 5 days with predatory cichlids *Parachromis managuensis* Günther, 1867 (37), using different methods of exposure and length of the stressor. Other species including Eurasian perch *Perca fluviatillis* Linnaeus, 1758 and rainbow trout *Oncorhynchus mykiss* Walbaum, 1792 showed habituation to mechanical stress after 8 weeks (60). In contrast, studies on catfish *Rhamdia quelen* Quoy and Gaimard, 1824 did not observe habituation after 3 weeks of exposure to mechanical stress (61). Thus, habituation to predatory presence in a time perspective, should be considered species specific. One might predict that cleaner fish and other species that partly include ectoparasites in their diet, have shorter habituation to predatory client fish, or share comparable behavioural and physiological traits that distinguish them from pure predator vs. prey interaction. The use of cortisol as a stress marker has limitations that should be taken into account when interpreting measurements of lumpfish tested in the following experiment. All measurements were done during afternoon, from 12:00 p.m. to 14:00 p.m. to avoid sampling bias. Thus, measured elevation of plasma cortisol might have looked different if trials had been conducted at a different time during the day or the season (62). In addition, Ellis et al. (62) reviewed cortisol in relation to fish welfare, and highlighted limitations on dose- and context dependency when analyzing cortisol responses in different fish species. In fish farming, stress is both multifactorial and unavoidable and the observed stress responses in naive lumpfish to Atlantic salmon should be accounted for as one such stressor when adding up all factors.

### Experimental Setup

In aquaculture, lumpfish are commonly deployed in sea cages with Atlantic salmon, while in this study the sequence of fish added to the tank was contrary with lumpfish already present when Atlantic salmon were added to the experimental tank. The study design made it possible to measure stress responses from Atlantic salmon introduction only, thus avoiding disruptive stressors including transportation and handling. Sea cages provided a larger and deeper water volume and exposed experienced fish to other stimuli that could have influenced how these individuals responded during the experiment. Thus, we cannot exclude the possibility that experience–other than exposure to predatory salmon–prior to the experimental testing, caused the difference in behaviour and stress between the two groups. However, the results showed that naive (kept in a tank) and not experienced lumpfish (kept in a sea cage) demonstrated increased stress (swimming behaviour, interspecies distance and cortisol levels) after the introduction of Atlantic salmon. This is the opposite of expected if the measured differences in stress level were a result of different experience (tank vs. cage), since the experimental trials were run in tanks, and not in cages. Moreover, the experiment revealed minimal differences in swimming behaviour between naive and experienced lumpfish during the first 10 min before salmon were introduced into the experimental tank, and the difference occurred the first minute after the introduction of salmon. These findings suggested that Atlantic salmon—and not the variation in environment (tank vs. cage)—caused a different response between the lumpfish groups.

## Conclusion

The following study suggest three measurements of habituation in experienced lumpfish after interactions with Atlantic salmon in commercial scale sea cages. In the experimental tank, analysis of naive lumpfish exposed to Atlantic salmon revealed increased swimming activity, increased interspecies distance, and elevated plasma cortisol concentrations. In comparison, experienced lumpfish showed no change in swimming activity when reintroduced to Atlantic salmon, shorter interspecies distance, and additional desensitized physiological stress responses depicted from significant lower plasma cortisol levels. Innate predatory stress responses in naive lumpfish are likely to influence their behaviour during first interactions with farmed Atlantic salmon, and the length of the habituation period should be taken account for when lumpfish are introduced to commercial use to improve welfare for the species. Further studies will focus on social behaviour in lumpfish, stress induced from specific predator sensory cues, and how knowledge on habituation could become more applicable in the aquaculture industry.

## Data Availability

The datasets generated for this study are available on request to the corresponding author.

## Ethics Statement

The study was conducted according to the Animal Welfare Act (LOV-2009-06-19-97) and the Norwegian law on Regulation of Animal Experimentation (FOR-1996-01-15-23). Handling of live fish was managed by personnel with FELASA-C course, based on the policies by the Federation of European Laboratory Animal Science Association. Lumpfish and Atlantic salmon used during the field experiment were assigned to project FDU 7835, accepted by the Norwegian Food Safety Authority under the regulation of the Research Animal Act (FOR-2015-06-18-761).

## Author Contributions

This work was part of a Ph.D. titled Habituation and learning in lumpsuckers during interaction with Atlantic salmon, with focus on behaviour and physiology involving the main author. All co-authors collaborated with the preparation of the manuscript and are fully responsible for its contents.

### Conflict of Interest Statement

The authors declare that the research was conducted in the absence of any commercial or financial relationships that could be construed as a potential conflict of interest.
